# Willingness to accept herpes zoster vaccines and the influencing factors in China

**DOI:** 10.1186/s12879-022-07840-2

**Published:** 2022-11-26

**Authors:** Binshan Jiang, Qing Wang, Zhenzhong Wang, Yunshao Xu, Tao Yang, Weizhong Yang, Mengmeng Jia, Luzhao Feng

**Affiliations:** 1 School of Population Medicine and Public Health, Chinese Academy of Medical Sciences and Peking Union Medical College, Beijing, 100730 China; 2 Chinese Academy of Medical Sciences and Peking Union Medical College, Beijing, 100730 China; 3 Peking Union Medical College Education Foundation, Beijing, 100730 China

**Keywords:** Herpes zoster, Herpes zoster vaccine, Willingness, Influencing factor, Aged, China

## Abstract

**Background:**

Herpes zoster increases the burden on the elderly in an aging society. Although an effective vaccine licensed by China Food and Drug Administration in 2019 was introduced into the market in June 2020, the willingness and influencing factors of herpes zoster vaccines in Chinese adults ≥ 50-years-old during coronavirus disease-2019 pandemic are yet to be elucidated.

**Methods:**

An online questionnaire survey was conducted using a simple random sampling method in October 2021 for viewers of the broadcast program. A binary logistic regression and multiple response analysis were conducted for herpes zoster vaccine and vaccination willingness. Pareto’s graphs were plotted to present the multiple-choice questions of influencing factors.

**Results:**

A total of 3838 eligible participants were included in this study. Among them, 43.02% intended to be vaccinated, including 10.34% self-reported about receiving at least one shot of shingles vaccine, 30.22% declined, and 26.76% were hesitant. This population comprised a large proportion of middle-aged and older people (≥ 50-years-old) who have not experienced an episode of herpes zoster (54.98%) or are unaware of the virus (33.22%). The strongest determinants of vaccine hesitancy among older people were education background of Master’s degree or above compared to senior high or equivalent and below, personal monthly income < 3000 RMB compared to 3000–5999 RMB, and living in a rural area.

**Conclusions:**

The willingness to get shingles vaccines can be improved further. Professional education and credible recommendation might prompt the elderly to improve their willingness and reassure them of the safety and efficacy of the vaccine. Also, accessibility and affordability should also be improved in the future.

**Supplementary Information:**

The online version contains supplementary material available at 10.1186/s12879-022-07840-2.

## Background

The primary infection of varicella-zoster virus (VZV) usually occurs in childhood and manifests as chickenpox, which subsequently remains dormant for a prolonged period in the spinal dorsal root ganglion or cranial ganglion of the spinal cord. When the corresponding immune response in the body drops below the critical threshold, VZV reactivates the infectious skin disease of herpes zoster (HZ) [1].

The aging of the population increases the incidence of HZ worldwide, especially Asia [2]. Studies have shown that the incidence of HZ is 3–5/1000 person-years in the general global population [3, 4] and 2.9–5.8/1000 person-years in 50-year-old individuals in mainland China [5]; this rate of incidence is increasing by 2.5–5.0% every year [6, 7]. In addition to skin damage, HZ is often accompanied by neuropathic pain (PHN), which affects the quality of life of patients as well as the loss of self-care ability. The increasing age gradually decreases the VZV-specific cellular immune function, which elevates the morbidity, hospitalization rate, and mortality of HZ [3, 4, 6]. The mean age of onset is 59.4, and 68% of HZ cases are individuals ≥ 50-years-old [1]. Thus, preventing HZ is vital to improving the quality of life of the elderly while relieving the healthcare systems and social pressure.

Two types of herpes zoster vaccines (HZVs), licensed globally, are the most effective methods to preventing HZ and the related complications. One is a recombinant zoster vaccine (RZV) available in China since 2020. Several studies have shown that RZV has a high and lasting protective effect in Asian patients ≥ 50-years-old (95.55%) [7, 8], people with various underlying diseases (84.5–97.0%) [9], and those infected with HZ (90.2%) [10]. However, only two studies have reported the willingness of Shanghai residents towards HZV [11, 12], and no statistical analysis of HZV uptake rate in the first year after its release is yet available. Nonetheless, the knowledge and confidence about vaccines, awareness of infection risk, service accessibility, and the number of underlying diseases are identified as the factors influencing the willingness to vaccination [11–13]. Compared to other vaccines applied in the aging Chinese population, disposable income was related to self-paid HZV [13]. Nation-wide data on the willingness towards HZV and the influencing factors in the high-risk population aged ≥ 50-years-old are crucial to improving the utility of HZV in HZ prevention.

Recently, the coronavirus disease-2019 (COVID-19) pandemic has brought about unprecedented positive changes in the public’s understanding and attitude towards vaccines. Some studies have proposed that the public’s demand for flu and pneumonia vaccines has increased [14–16]. Since more than 200 cities have been officially listed with the availability of HZV to prevent HZ in individuals ≥ 50-years-old, we expected a rise in HZV uptake. However, the public’s willingness to be vaccinated against HZ is still unclear in China. Thus, the present study aimed to evaluate the public acceptance of HZV and estimate the determinants of HZ vaccination uptake in the post-pandemic era, which would guide the feasible steps.

## Methods

### Participants and data collection

A live health promotional event with the theme of “Vaccines and Health in the Elderly” was broadcast by the People’s Daily Health platform and available on more than ten online platforms simultaneously, like Tencent Video, Sohu Video, Toutiao. During the event, experts in the field of public health, dermatologists, and respiratory physicians were invited to promote knowledge about the prevention and treatment of herpes zoster, influenza, and pneumococcal pneumonia in the elderly, with emphasis on the role of related vaccines. The audiences who watched the live broadcast were invited to participate in our online survey about their attitude toward the influenza vaccine and HZV on the www.wjx.cn (URL) portal. The link of the survey was pinned on the comment box and audiences can start answering the survey at any time during the broadcast. Whether they participated or not in the vaccination had no effect on watching the live broadcast. Informed consent was obtained at the beginning of the survey. The audiences who refused to complete the questionnaire exit the survey link automatically.

### Measurements and variables

The questionnaire was in Chinese, and participants were requested to answer about the demographic information (gender, age group, educational level, personal monthly income, employment status or professional, living area, and region). Participants ≥ 50-years-old had access to additional questions about (1) history of underlying diseases and HZ (“Do you have any following chronic diseases,” and “Have you ever experienced an episode of HZ?”); (2) willingness of HZV and reasons. The willingness to be vaccinated for HZ was assessed with the question, “Would (Have) you get(gotten) vaccinated against HZ?”, followed by the response options “Yes, I have already gotten”, “Yes, but I have not gotten yet”, “No”, and “Not sure” The survey items are listed in Additional file 1.

### Quality control

All data provided to the investigators were de-identified, and the participants were anonymous. The quality control was conducted through the survey platform, wherein the respondents were invited to fill in the questionnaire voluntarily. Each respondent identified by the same device, user name, and IP address could fill in the questionnaire only once. After the responses were submitted, each respondent was screened by the investigators to retain the valid questionnaires. Those that failed the attention check answered all the questions the same or provided cyclical responses, who finished the questionnaire within 60 s, and those < 18-years-old were unqualified. Also, the consistency between the IP address and the selected region was verified to filter out random answers. After eliminating the invalid responses through data filtering, 10,223 questionnaires were valid (rate of 83.3%), with 3838 respondents ≥ 50-years-old.

### Statistical analysis

All categorical variables were summarized using frequencies and percentages. The univariate analysis included chi-square tests or binary logistic regression; P-values < 0.05 indicated a statistically significant difference. Univariable and multivariable logistic regression was used to examine the predictive indicators of HZ vaccination intention in the elderly. Also, odds ratios (OR) and 95% confidence intervals (CI) were calculated. The dependent variable was the intention of HZ vaccination, including “Intent to be vaccinated” (whether have already gotten HZV), “Not intended to be vaccinated,” and “Not sure.” The demographic characteristics, including gender, age group, educational attainment, professional, personal monthly income, area, history of underlying diseases, and HZ history, were included in the multivariable analysis.

The cumulative percentages for multiple-choice questions were calculated, and Pareto’s charts were plotted, offering votes in descending order by columns and cumulative by the line.

Microsoft Excel (version 2019, Redmond, WA, USA) and IBM SPSS Statistics (version 19.0, Armonk, NY, USA) were used for data cleaning and analyses.

## Results

The health promotional event was watched by 2.77 million people, covering 31 provinces of mainland China. During the broadcast, a total of 3988 elders responded to our survey, and 150 (3.76%) were exclude after quality control. Among the 3838 participants included in our analysis, 65.69% were 50–59-years-old, and about 49.37% were females. Approximately 49.53% of them had less than a Bachelor’s degree, 43.04% were not working or were retired, and about 67.07% earned their monthly income by pension (3000–5999 RMB). The majority of the participants (64.88%) were from urban areas. About 34.86% were accompanied by self-reported underlying diseases. Furthermore, only 11.80% (453/3838) reported a personal history of HZ; among them, 68/453 (15.01%) had been vaccinated. The participant characteristics are summarized in Table 1.

**Table 1:** Participant characteristics and their attitude towards HZV (n = 3838)

Characteristic	N	%*
Gender		
Female	1895	49.37
Male	1943	50.63
Age group (years)		
50–59	2521	65.69
60–69	811	21.13
≥ 70	506	13.18
Educational attainment		
Senior high or equivalent and below	1901	49.53
College/Bachelor’s degree	1388	36.16
Master’s degree or above	549	14.30
Professional		
Not working or retired	1652	43.04
Health workers	268	6.98
Government agencies, enterprises/institutions staff	684	17.82
Business or service industries, such as transportation and food	684	17.82
Education (except medical teachers)	270	7.03
Others	280	7.30
Personal monthly income (RMB)		
< 3000	673	17.54
3000–5999	2574	67.07
6000–9999	517	13.47
≥ 10,000	74	1.93
Area		
Urban area	2490	64.88
Rural area	1348	35.12
Underlying diseases		
No	1338	34.86
Yes	2500	65.14
Have you ever experienced an episode of herpes zoster?		
No	2110	54.98
Yes	453	11.80
Not sure	1275	33.22
Would (Have) you get (gotten) vaccinated against herpes zoster?		
Yes, I have already gotten	397	10.34
Yes, but I have not gotten yet	1254	32.67
No	1160	30.22
Not sure	1027	26.76

Underlying diseases include chronic respiratory disease, cardiovascular disease, hypertension, diabetes, cancer, immune system disease, and other self-administrated diseases

*Percentages may not total to 100 owing to rounding

Overall, 1651/3858 (43.02%) of responders intended to be vaccinated, 1027/3838 (26.76%) were not sure whether they would be vaccinated, and 1160/3838 (30.22%) did not intend to be vaccinated. The participants associated with a higher chance of responding “no” or “not sure” vs. “yes” had at a higher education level, with a personal income of 6000–9999 RMB, but the personal history of HZ was not explicated. On the other hand, the participants who responded “no” were likely from a rural environment (Table 2).

**Table 2:** Intent to be vaccinated based on participant characteristics

Participant characteristic	Intent to be vaccinated, *n* (%)*			Single-factor logistic regression P-value		Multivariate logistic regression OR (95% CI)	
	Yes (n = 1651)	No (n = 1160)	Not sure (n = 1027)	No vs. yes	Not sure vs. yes	No vs. yes	Not sure vs. yes
Gender							
Female	818 (43.17)	552 (29.13)	525 (27.70)	0.306	0.428	Reference	Reference
Male	833 (42.87)	608 (31.29)	502 (25.84)			1.03 (0.88–1.20)	0.91 (0.77–1.06)
Age group (years)							
50–59	1064 (42.21)	777 (30.82)	680 (26.97)	0.326	0.078	Reference	Reference
60–69	375 (46.24)	238 (29.35)	198 (24.41)			0.90 (0.67–1.21)	0.86 (0.64–1.16)
≥ 70	212 (41.90)	145 (28.66)	149 (29.45)			0.97 (0.70–1.34)	1.16 (0.84–1.60)
Educational attainment							
Senior high or equivalent and below	876 (46.08)	526 (27.67)	499 (26.25)	**0.000**	**0.035**	Reference	Reference
College/Bachelor’s degree	568 (40.92)	450 (32.42)	370 (26.66)			1.16 (0.98–1.37)	1.05 (0.88–1.26)
Master’s degree or above	207 (37.70)	184 (33.52)	158 (28.78)			**1.29 (1.02–1.63)**	1.25 (0.98–1.59)
Professional							
Not working or retired	736 (44.55)	481 (29.12)	435 (26.33)	0.187	0.705	Reference	Reference
Health workers	110 (41.04)	85 (31.72)	73 (27.24)			0.96 (0.66–1.39)	1.00 (0.68–1.47)
Government agencies, enterprises/institutions staff	301 (44.01)	189 (27.63)	194 (28.36)			0.76 (0.56–1.04)	0.96 (0.71–1.31)
Business or service industries, such as transportation and food	276 (40.35)	221 (32.31)	187 (27.34)			0.96 (0.71–1.31)	0.99 (0.72–1.36)
Education (except medical teachers)	115 (42.59)	93 (34.44)	62 (22.96)			1.00 (0.69–1.44)	0.80 (0.54–1.20)
Others	113 (40.36)	91 (32.50)	76 (27.14)			1.08 (0.75–1.57)	1.08 (0.74–1.59)
Personal monthly income (RMB)							
3000–5999	1026 (39.86)	858 (33.33)	690 (26.81)	**0.000**	**0.000**	Reference	Reference
< 3000	390 (57.95)	121 (17.98)	162 (24.07)			**0.39 (0.31–0.49)**	**0.64 (0.52–0.80)**
6000–9999	202 (39.07)	159 (30.75)	156 (30.17)			0.95 (0.76–1.19)	1.16 (0.92–1.46)
≥ 10,000	33 (44.59)	22 (29.73)	19 (25.68)			0.82 (0.47–1.43)	0.86 (0.48–1.53)
Area							
Urban area	1102 (44.26)	709 (28.47)	679 (27.27)	**0.002**	0.712	Reference	Reference
Rural area	549 (40.73)	451 (33.46)	348 (25.82)			**1.24 (1.06–1.45)**	1.01 (0.85–1.19)
Underlying diseases							
No	1067 (42.68)	755 (30.20)	678 (27.12)			Reference	Reference
Yes	584 (43.65)	405 (30.27)	349 (26.08)	0.802	0.463	0.97 (0.82–1.13)	0.93 (0.78–1.09)
Have you ever experienced an episode of herpes zoster?							
No	935 (44.31)	618 (29.29)	557 (26.40)	**0.003**	**0.019**	Reference	Reference
Yes	217 (47.90)	125 (27.59)	111 (24.50)			0.87 (0.68–1.11)	0.84 (0.65–1.09)
Not sure	499 (39.14)	417 (32.71)	359 (28.16)			1.14 (0.96–1.35)	1.15 (0.96–1.37)
Total	1651 (43.02)	1160 (30.22)	1027 (26.76)				

*Percentages may not total to 100 owing to rounding

Bolded text indicates statistically significant (P-values < 0.05)

Underlying diseases include chronic respiratory disease, cardiovascular disease, hypertension, diabetes, cancer, immune system disease, and other self-administrated diseases

After adjusting for demographic characteristics, factors that were independently associated with vaccine hesitancy (response of “no” or “not sure”) include personal monthly income < 3000 RMB. Participants from a rural setting had a 1.24-fold higher relative likelihood of responding “no” *vs*. “yes” compared to those living in the urban areas. Participants with a Master’s degree or above responded “no” when asked about intent to be vaccinated, but not with “not sure.” The other characteristics, such as gender, age strata, professional, and underlying chronic diseases, were associated with vaccination intent but did not achieve statistical significance consistently for both response categories (“no” and “not sure”). Some educational levels and personal history of herpes zoster were not significantly associated with HZ vaccination intent after adjustment for the characteristics listed in Table 2.

**Fig. 1:**
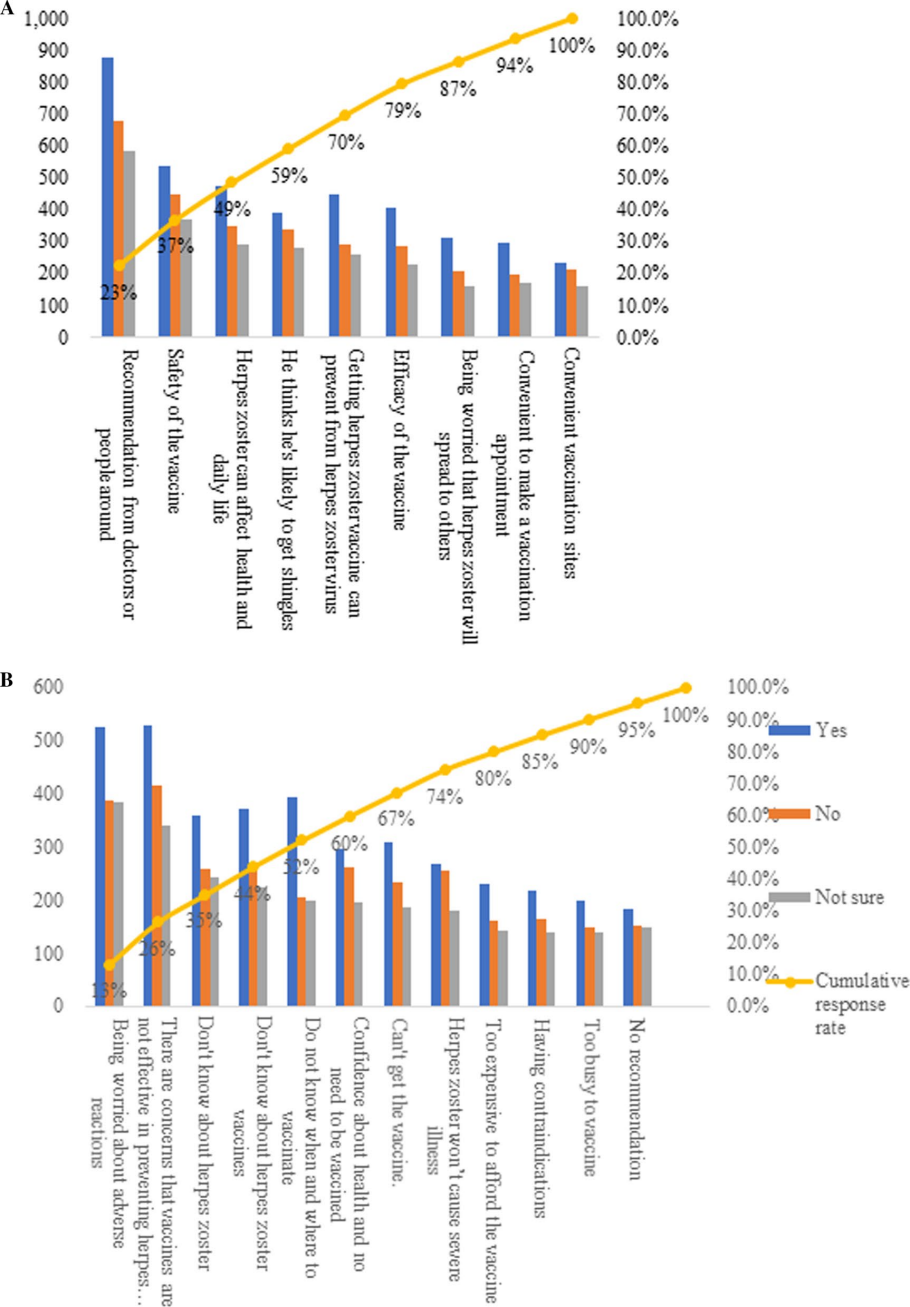
Multiple response factors influencing the willingness to be vaccinated against HZ for the elderly in China. We investigated why respondents would be willing and unwilling to get vaccines regardless of whether they expressed willingness or hesitancy. **A** For willing and unwilling vaccinators, the reasons that prompted them to be vaccinated. **B** For willing and unwilling vaccinators, factors influencing their unwillingness to be vaccinated

Despite their intent regarding HZ vaccination, the participants were asked to offer their main reasons affecting their acceptance or denial. The three most common reasons cited by participants who intended or were hesitant to be vaccinated included being recommended, believing in the safety of the vaccine, and being worried about the harm inflicted by HZ on their health and activities. For all participants, recommendations were the leading factor promoting willingness. Notably, the efficacy and the adverse reactions were the top two concerns that impede vaccine uptake for individuals with intent and no intent to be vaccinated. Compared to those willing to get the vaccination, which is concerned about the time and place of vaccination, unawareness of HZ was more powerful in explaining HZ vaccine hesitancy (Fig. 1).

Figure 1A depicts that being recommended, the safety of the HZV, the severity of HZ, susceptibility of HZ, HZV protecting HZ, and the efficacy of the HZV account for nearly 80% of the factors to the intention or vaccination. 28.4/80% of intention is attributed to the recommendation. Among the 12 reasons accounted for staying away from HZV shown in Fig. 1B, 80% were due to being worried about adverse reactions and effectiveness of HZV, unawareness of HZ and HZV, lack of HZV vaccination-related information, inaccessibility or unavailability of HZV, and unaffordability of the vaccine.

## Discussion

One year after being introduced into the domestic market, the first vaccine used against HZ in mainland China, HZV has now been supplied in over 200 cities. The number of HZ vaccinees remains very low, which is similar to the situation in the USA in the initial 2–3 years when HZV was recommended to the public in 2006. Since the evidence related to the HZ vaccine uptake rate is limited [17], we did not consider the vaccination rate of HZV as the outcome variable; about 50% of participants showed hesitancy for HZV. The present study identified the demographic factors related to HZV willingness and reasons for vaccination hesitancy. The public presented common main factors irrespective of their attitudes.

In the current survey, 43.02% of participants intended to be vaccinated, 30.22% declined, and 26.76% were hesitant. This rate (43.02%) was in accordance with a previous study in Shanghai (49.64%) before HZV was available [12], but the willingness to get HZV was 26% higher compared to another Shanghai study conducted in late 2020 with the elderly’s intention of HZ vaccination (16.57%) [11]. This difference could be attributed to the different populations, methods of response, and the positive impact on all vaccines due to COVID-19 vaccination. However, a huge gap between willingness and behavior in the vaccination is notable since < 25% (397/1651) of willing participants have received HZV.

As expected, participants from rural areas showed strong unwillingness largely due to access and affordability issues concerning HZV. We also found that low personal income has a strong influence on vaccination hesitancy since HZV is a self-paid vaccine in China. However, those with personal monthly incomes < 3000 RMB expressed a strong willingness to HZV. This unexpected result hinted that the unanticipated COVID-19 pandemic might have a significant impact on the normal life of the underclass, and the acceptance of COVID-19 vaccine can be split over into the HZV, after the free COVID-19 vaccination program in China. In addition, HZV is relatively new in China, it is possible that most people have no clear idea how much it cost to be vaccinated against HZ. Further studies are needed to support this finding, especially for studies on their willingness to pay. To translate the strong willingness into actions, multipronged social support strategies should be executed; for instance, introducing HZV into insurance or promoting free vaccines for high-risk patients [18]. Several studies have verified that the national or government-supported vaccine program and insurance cost-sharing help in increasing the acceptance and actual uptake [19]. Accumulating evidence indicated that the COVID-19 pandemic has changed the public attitude positively regarding the vaccines not limited to COVID-19 [15, 16], which can be considered remarkable among low-income populations in our study. This finding necessitates further studies to substantiate this conclusion in the future.

The uncommon fact consistent with the results of Zein et al. was that the odds of refusal of getting HZ vaccination increased slightly with high education level [20]. Graduate degree holders (Master’s and Ph.D.) presented a 1.29-fold higher probability of refusal to get HZV than participants with senior high school or lesser diploma (OR 1.29, 95% CI: 1.02–1.63). Although the statistical significance was only borderline, indicating that the refusal of the vaccine was not arbitrary but may be associated with specific education. Higher education experience may boost their self-efficacy associated with complacency and determines the degree of hesitancy [21]. Such finding informs practitioners that it should not to be assumed that higher education is associated with better health literacy. Tailored health educational programs about HZV are needed for elders with different educational background.

To achieve satisfactory coverage, the actions that can prompt the elderly need to be considered seriously. The recommendation from other people was chosen as a decisive factor among those ≥ 50-years-old [22] since they are isolated compared to younger generations with respect to accurate, timely, and professional health knowledge. In practice, under American Advisory Committee on Immunization rapid approval, two-dose completion of RZV within one year exceeds that for other vaccine series used in American and Canadian elderly (80% and 74.9%, respectively) [23]. Given the importance of receiving recommendations for the HZ vaccine, people around them play a critical role in this process. Enhancing the clinicians’ responsibilities and abilities to recognize their patients’ demands [24], carrying out community education, and informing their grown-up children could potentially increase the chance of recommendations to the elderly [17]. Especially, healthcare providers are the primary personnel who may offer immunization information and recommendation. Their role is that of a population health practitioner focusing not only on the diagnosis and treatment but on disease prevention and health promotion. Typically, the recommendation or vaccination history of other vaccines, such as like influenza and pneumococcal infection, are beneficial to HZ vaccination; also, speaking about immunization is beneficial. Evidentially, 69.5% of the refusals reversed to accept HZV following the physician’s recommendation in South Korea [25]. Moreover, confirming the safety of the vaccine and perceived severity and susceptibility to HZ prompted the elders to get HZV. These elements may help the healthcare providers to train their communication skills and content.

In our survey, 56.98% of participants might not have the HZV; among them, 53.04% definitely did not have the vaccine. Therefore, we confirmed the target population’s obstacles to vaccination. These results might be useful in prioritizing the targeted actions aiming to achieve satisfied coverage rates. Fear of adverse reactions and concerns of effectiveness belongs to the confidence category and get more consensus than other factors, such as lack of knowledge and information. Providing health education and improving health literacy is an urgent requisite for elders than providing those with vaccination-related information, which should be taken as the second step. Although people have access to the vaccine, they would not accept it unless they have ample confidence in the safety and efficiency of the HZV. To alleviate the asymmetric information, both scientific and experience-based evidence needs to be presented to the elderly. Various ways of health education should be adopted as information sources for the Internet-savvy elderly, such as playing short videos on TikTok, broadcasting public health announcements on social media, and organizing peer education in their familiar community.

The strengths of this study are that we investigate the main reasons for being willing or not willing to be vaccinated in a large sample size of Chinese elderly. Nevertheless, the limitations cannot be ignored. First, the cross-sectional nature of the present study does not allow the comparison of changes in HZV intention from before to after the introduction of the vaccine in China. This could be carried out only by indirect comparison with limited published studies. Second, the online survey made it impossible for us to recruit those who have no access to the Internet and those who had difficulties reading or using mobile phones. Collecting data from the People’s Daily Health media platform, where the adopters focus on their health, may induce selection bias and volunteer bias and lead to the overestimation of the coverage rate and the willingness to receive HZV. To weaken these unavoidable biases, our results emphasized the factors rather than numerical calculations.

## Conclusions

The current study demonstrated the nonoptimal HZV coverage in at-risk populations despite a fairly positive perception of HZVs and confirmed that physicians are on the front lines to suggest and recommend these vaccinations, especially in the current COVID-19 pandemic. Vaccine hesitancy is multifaceted and might require multisectoral and multidisciplinary strategies to engage in conversations addressing public concerns. To stimulate HZV acceptance in the Chinese elderly, three objectives must be achieved: (a) professional education and credible recommendation to resolve information privatization problems; (b) matching supply with demand to resolve the inaccessible problem; (c) implementing insurance programs to resolve the affordable problem. As the COVID-19 widely vaccination highlighted the value of vaccines than ever before, efforts are being made to boost the confidence of the aging society in vaccination.

## Supplementary Information

**Additional file 1: Table S1.** Top three reasons for willing to be vaccinated against herpes zoster for the elderly in China (n=9482). **Table S2.** Top three reasons for not willing to be vaccinated against herpes zoster for the elderly in China (n=9805). **Table S3.** Sensitivity analysis of intent to be vaccinated based on participant characteristics.

## Data Availability

Anonymized individual-level data and datasets generated or analyzed during the current study are available for researchers interested in similar studies. Please contact Mengmeng Jia (jiamengmeng@cams.cn) and Luzhao Feng (fengluzhao@cams.cn).

## Abbreviations

CI: Confidence intervals
HZ: Herpes zoster
HZV: Herpes zoster vaccine
OR: Odds ratios
PHN: Neuropathic pain
RZV: Recombinant zoster vaccine
VZV: Varicella-zoster virus
