# Supplementary material for: Willingness to accept herpes zoster vaccines and the influencing factors in China
Source: BMC Infect Dis. 2022 Nov 26;22:888. doi: 10.1186/s12879-022-07840-2 (PMC9701420; doi:10.1186/s12879-022-07840-2)
Supplement: Supplementary file 1 — Additional file 1: Table S1. Top three reasons for willing to be vaccinated against herpes zoster for the elderly in China (n=9482). Table S2. Top three reasons for not willing to be vaccinated against herpes zoster for the elderly in China (n=9805). Table S3. Sensitivity analysis of intent to be vaccinated based on participant characteristics. [file 12879_2022_7840_MOESM1_ESM.docx]

**Willingness to accept herpes zoster vaccines and the influencing factors in China**

Binshan Jiang^a^, Qing Wang^a^, Zhenzhong Wang^a^, Yunshao Xu^a^, Tao Yang ^b,c^, Weizhong Yang^a^, Mengmeng Jia ^a, *^, Luzhao Feng^a, *^

a. School of Population Medicine and Public Health, Chinese Academy of Medical Sciences & Peking Union Medical College, Beijing 100730, China

b. Chinese Academy of Medical Sciences & Peking Union Medical College, Beijing 100730, China

c. Peking Union Medical College Education Foundation, Beijing 100730, China

* Corresponding Authors. [jiamengmeng@cams.cn](mailto:jiamengmeng@cams.cn) (M. M. Jia); [fengluzhao@cams.cn](mailto:fengluzhao@cams.cn) (L. Z. Feng)

Phone number: 010-69155957

**Appendix**

**Questionnaire on the willingness to accept herpes zoster vaccines**

**Dear Sir/Madam,**

**In order to understand what the public is doing about vaccination in China, the School of Population Medicine and Public Health of Peking Union Medical College is conducting this public service survey. It will take you about several minutes to answer. No personally identifiable information is involved in the questionnaire, and the data collected will be used for scientific research only. Please answer as you see fit. Your answers are important for the development of a reasonable vaccination strategy and the promotion of a healthier China built! Thanks for your cooperation!**

**Are you willing to accept this survey?**

Yes.

No. (End, exit the page)

**Your gender**

Female

Male

**How old are you?**

50-59y

60-69y

≥70y

**What is your highest educational attainment?**

Senior high or equivalent and below

College/Bachelor’s degree

Master’s degree or above

**What is your professional status?**

Not working or retired

Health workers

Government agencies, enterprises/institutions staff

Business or service industries such as transportation and food

Education (except medical teachers)

Others

**Which income level are you at each month (RMB)?**

＜3 000

3 000-5 999

6 000-9 999

≥10 000

**Where do you live now?**

City Urban area

Rural area

**Do you have any following chronic diseases?**

**chronic respiratory disease, cardiovascular disease, hypertension, diabetes, cancer, immune system disease, or other self-administrated diseases**

None of these.

Yes.

**Have you ever experienced an episode of herpes zoster?**

No.

Yes.

I cannot remember.

**Would (Have) you get (gotten) vaccinated against herpes zoster?**

Yes, I have already gotten.

Yes, but I have not gotten yet.

No.

Not sure.

**What made or would make you intend to get shingle vaccines? Please choose not more than three main reasons from following statements.**

Being recommended by people around or doctors.

Getting herpes zoster vaccine can prevent from herpes zoster virus

Efficacy of the vaccine.

Safety of the vaccine.

He thinks he’s likely to get herpes zoster.

Herpes zoster can affect health and daily life.

Being worried that herpes zoster will spread to others.

Convenient to make a vaccination appointment.

Convenient vaccination sites.

**What made or would make you refuse to get shingle vaccines? Please choose not more than three main reasons from following statements.**

Don't know about herpes zoster.

Don't know that herpes zoster vaccines exist.

Do not know when and where to vaccinate.

There are concerns that vaccines are not effective in preventing herpes zoster virus.

Being worried about adverse reactions.

Confidence about health and no need to be vaccinated.

Herpes zoster won’t cause severe illness.

Can't get the vaccine.

Inconvenient vaccination sites

Too expensive to afford the vaccine.

Having contraindications.

Too busy to vaccine.

No recommendation.

We investigated the reasons why respondents were willing and unwilling to vaccinate regardless of whether they were willing to vaccinate or not.

| **Table S1. Top three reasons for willing to be vaccinated against herpes zoster for the elderly in China (n=9482).** | | |
| --- | --- | --- |
| **Reasons for vaccination*** | intention (n, %) | hesitancy (n, %) |
| Being recommended by people around or doctors. | 878 (22.88) | 1264 (32.93) |
| Getting herpes zoster vaccine can prevent from herpes zoster virus | 446 (11.62) | 552 (14.38) |
| Efficacy of the vaccine. | 404 (10.53) | 517 (13.47) |
| Safety of the vaccine. | 536 (13.97) | 820 (21.37) |
| He thinks he’s likely to get herpes zoster. | 390 (10.16) | 618 (16.10) |
| Herpes zoster can affect health and daily life. | 472 (12.30) | 638 (16.62) |
| Being worried that herpes zoster will spread to others. | 311 (8.10) | 365 (9.51) |
| Convenient to make a vaccination appointment. | 294 (7.66) | 369 (9.61) |
| Convenient vaccination sites. | 234 (6.10) | 374 (9.74) |
| *These reasons are not mutually exclusive.  For this question, percentages do not total to 100 because each responder could choose 1 to 3 options. | | |

| **Table S2. Top three reasons for not willing to be vaccinated** **against herpes zoster for the elderly in China (n=9805).** | | |
| --- | --- | --- |
| **Reasons for not being vaccination*** | intention (n, %) | hesitancy (n, %) |
| Don't know about herpes zoster. | 360 (9.38) | 502 (13.08) |
| Don't know that herpes zoster vaccines exist. | 373 (9.72) | 484 (12.61) |
| Do not know when and where to vaccinate. | 394 (10.27) | 406 (10.58) |
| There are concerns that vaccines are not effective in preventing herpes zoster virus. | 530 (13.81) | 756 (19.70) |
| Being worried about adverse reactions. | 527 (13.73) | 772 (20.11) |
| Confidence about health and no need to be vaccinated. | 297 (7.74) | 460 (11.99) |
| Herpes zoster won’t cause severe illness. | 269 (7.01) | 438 (11.41) |
| Can't get the vaccine. | 309 (8.05) | 420 (10.94) |
| Inconvenient vaccination sites | 202 (5.26) | 281 (7.32) |
| Too expensive to afford the vaccine. | 230 (5.99) | 301 (7.84) |
| Having contraindications. | 218 (5.68) | 303 (7.89) |
| Too busy to vaccine. | 200 (5.21) | 289 (7.53) |
| No recommendation. | 184 (4.79) | 300 (7.82) |
| *These reasons are not mutually exclusive.  For this question, percentages do not total to 100 because each responder could choose 1 to 3 options. | | |

| **Table S3. Sensitivity analysis of intent to be vaccinated based on participant characteristics.** | | | |
| --- | --- | --- | --- |
| **Participant Characteristic** | | \| Multivariate logistic regression  OR (95% CI) \| \| --- \| | |
|  |  | No *vs*. Yes | Not Sure *vs*. Yes |
| **Gender** | |  |  |
|  | Female | Reference | Reference |
|  | Male | 1.00 (0.85-1.18) | 0.89 (0.75-1.05) |
| **Age group (years)** | |  |  |
|  | 50–59 | Reference | Reference |
|  | 60–69 | 0.97 (0.72-1.33) | 0.94 (0.69-1.29) |
|  | ≥70 | 0.97 (0.69-1.37) | 1.18 (0.84-1.65) |
| **Educational attainment** | |  |  |
|  | Senior high or equivalent and below | Reference | Reference |
|  | College/Bachelor’s degree | 1.14 (0.95-1.36) | 1.03 (0.86-1.24) |
|  | Master’s degree or above | 1.23 (0.96-1.57) | 1.18 (0.92-1.52) |
| **Professional** | |  |  |
|  | Not working or retired | Reference | Reference |
|  | Health workers | 0.97 (0.65-1.45) | 1.03 (0.68-1.54) |
|  | Government agencies, enterprises/institutions staff | 0.79 (0.57-1.09) | 1.00 (0.72-1.38) |
|  | Business or service industries, such as transportation and food | 1.01 (0.73-1.39) | 1.05 (0.75-1.46) |
|  | Education (except medical teachers) | 1.09 (0.74-1.62) | 0.89 (0.59-1.36) |
|  | Others | 1.14 (0.77-1.69) | 1.15 (0.77-1.72) |
| **Personal monthly income (RMB)** | |  |  |
|  | 3000–5999 | Reference | Reference |
|  | <3000 | **0.43 (0.34-0.55)** | **0.71 (0.57-0.90)** |
|  | 6000–9999 | 0.99 (0.78-1.26) | 1.21 (0.95-1.55) |
|  | ≥10,000 | 0.93 (0.51-1.69) | 0.98 (0.53-1.83) |
| **Area** | |  |  |
|  | Urban area | Reference | Reference |
|  | Rural area | **1.28 (1.08-1.51)** | 1.04 (0.87-1.24) |
| **Underlying diseases** | |  |  |
|  | No | Reference | Reference |
|  | Yes | 0.90 (0.76-1.06) | 0.86 (0.72-1.03) |
| **Have you ever experienced an episode of herpes zoster?** | |  |  |
|  | No | Reference | Reference |
|  | Yes | 0.97 (0.74-1.26) | 0.94 (0.71-1.23) |
|  | Not sure | 1.15 (0.96-1.38) | 1.15 (0.96-1.39) |
| **Total** | |  |  |
| * Percentages may not total to 100 owing to rounding.  Bolded text indicates statistically significant (P-values <0.05)  Underlying diseases include chronic respiratory disease, cardiovascular disease, hypertension, diabetes, cancer, immune system disease, and other self-administrated diseases. | | | |
